# Use of Free-Living Step Count Monitoring for Heart Failure Functional Classification: Validation Study

**DOI:** 10.2196/12122

**Published:** 2019-05-17

**Authors:** Jonathan-F Baril, Simon Bromberg, Yasbanoo Moayedi, Babak Taati, Cedric Manlhiot, Heather Joan Ross, Joseph Cafazzo

**Affiliations:** 1 Centre for Global eHealth Innovation Techna Institute University Health Network Toronto, ON Canada; 2 Institute of Biomaterials & Biomedical Engineering University of Toronto Toronto, ON Canada; 3 Ted Rogers Centre of Excellence in Heart Function University Health Network Toronto, ON Canada; 4 Toronto Rehabilitation Institute University Health Network Toronto, ON Canada; 5 Institute of Health Policy, Management and Evaluation Dalla Lana School of Public Health University of Toronto Toronto, ON Canada

**Keywords:** exercise physiology, heart rate tracker, wrist worn devices, Fitbit, heart failure, steps, cardiopulmonary exercise test, ambulatory monitoring

## Abstract

**Background:**

The New York Heart Association (NYHA) functional classification system has poor inter-rater reproducibility. A previously published pilot study showed a statistically significant difference between the daily step counts of heart failure (with reduced ejection fraction) patients classified as NYHA functional class II and III as measured by wrist-worn activity monitors. However, the study’s small sample size severely limits scientific confidence in the generalizability of this finding to a larger heart failure (HF) population.

**Objective:**

This study aimed to validate the pilot study on a larger sample of patients with HF with reduced ejection fraction (HFrEF) and attempt to characterize the step count distribution to gain insight into a more objective method of assessing NYHA functional class.

**Methods:**

We repeated the analysis performed during the pilot study on an independently recorded dataset comprising a total of 50 patients with HFrEF (35 NYHA II and 15 NYHA III) patients. Participants were monitored for step count with a Fitbit Flex for a period of 2 weeks in a free-living environment.

**Results:**

Comparing group medians, patients exhibiting NYHA class III symptoms had significantly lower recorded 2-week mean daily total step count (3541 vs 5729 [steps], *P*=.04), lower 2-week maximum daily total step count (10,792 vs 5904 [steps], *P*=.03), lower 2-week recorded mean daily mean step count (4.0 vs 2.5 [steps/minute], *P*=.04,), and lower 2-week mean and 2-week maximum daily per minute step count maximums (88.1 vs 96.1 and 111.0 vs 123.0 [steps/minute]; *P*=.02 and .004, respectively).

**Conclusions:**

Patients with NYHA II and III symptoms differed significantly by various aggregate measures of free-living step count including the (1) mean and (2) maximum daily total step count as well as by the (3) mean of daily mean step count and by the (4) mean and (5) maximum of the daily per minute step count maximum. These findings affirm that the degree of exercise intolerance of NYHA II and III patients as a group is quantifiable in a replicable manner. This is a novel and promising finding that suggests the existence of a possible, completely objective measure of assessing HF functional class, something which would be a great boon in the continuing quest to improve patient outcomes for this burdensome and costly disease.

## Introduction

Heart failure (HF), a global epidemic [1,2], is a complex chronic progressive condition associated with significant morbidity and mortality. HF is the leading cause of hospitalizations in the country, costing Canadians an estimated Can $3 billion annually [3]. From both a systems and patient-centered perspective, clinicians caring for patients with HF have a strong desire to reduce hospitalizations [3,4]. To do so, it is important for clinicians to be able to reliably assess disease progression and severity.

One of the ways in which HF is categorized is by the degree to which a patient’s left ventricle retains the ability to pump out the blood it receives—known as the left ventricular ejection fraction (LVEF) [5,6]. The degree to which ejection fraction (EF) is reduced can be an indicator of what part of, and to what degree, the heart has been damaged [5]. Practice guidelines recommend different interventional strategies according to the degree of preserved (or reduced) EF [5]. Broadly speaking, patients with an LVEF ≤40% are classified as suffering from a subtype of HF known as HF with reduced EF (HFrEF) [5,6]. Those with preserved EF are labeled as suffering from HF with preserved EF (HFpEF). Both subtypes are fairly common, with HFpEF comprising approximately 44% to 72% of cases, although it is difficult to make precise estimates as the exact LVEF cut-off for HFpEF versus HFrEF has varied over time and across geographic regions [6]. Nevertheless, current estimates indicate that HFpEF is starting to emerge as the most prevalent HF subtype (compared with HFrEF) in Canada and the United States, especially relative to the rest of the world [6,7].

Although decidedly more common in patients with HFrEF, the primary cause of HF overall is most commonly attributable to coronary heart disease (CHD): about 23% to 73% of patient cases depending on the study in question [8]. Hypertension (HT), often more associated with patients suffering from HFpEF, follows second as the hierarchy of competing common etiologies; of course, both CHD and HT commonly coexist in the same patient, which makes identifying the causal primacy of each condition difficult, especially as both CHD and HT are known to cause either type of HF [5,8]. For example, an analysis of patients in the well-known Framingham Heart Study showed that 63% of the 314 patients with HFrEF had CHD identified as the primary cause compared to 19% with HT identified as the primary cause [9]. In contrast, of the 220 patients with HFpEF, only 37% had CHD identified as the primary cause versus 36% with HT as the primary cause [9]. Of course, HF has many other known causes including valvular disease, congenital cardiac malformations, and pathogenic, nutritional, or toxicological causes, but CHD and HT are by far the most common [5].

As a result of the etiology of HF, in Canada, although not exclusively a disease of old age, HF prevalence and incidence increases sharply among Canadians aged 65 years and older, as expected from the high incidence and prevalence of cardiovascular disease (and CHD and HT in particular) among this subpopulation [4,10,11]. According to the Canadian Chronic Disease Surveillance System, in 2015, the crude prevalence rates among Canadian men aged between 40 and 49, 50 and 64, 65 and 79, and 80+ years were 0.34, 1.82, 7.07, and 20.02 (%), respectively, with slightly lower prevalence rates among women of the same age brackets at 0.23, 1.07, 4.65, and 17.92 (%), respectively [11]. In the last decade and a half of reported data (2000 to 2015), the age-standardized prevalence (among those aged 40 years and older) has also remained fairly constant, hovering around a mean (SD) of 3.07 % (SD 0.10 %^2^) for women and approximately 31% higher for men at 4.03 % (SD 0.09 %^2^) [11]. The incidence rate (for the same subpopulation), however, declined over the same period, from a peak of 952 to 612 (per 100,000) for men and from a peak of 714 to 459 (per 100,000) for women [11]. No data were recorded for those aged younger than 40 years [11].

One of the main manifestations of HF across populations is exercise intolerance [5,12]. As a result, apart from evaluating LVEF (among other biometrics), evaluating exercise intolerance forms an integral part of HF care and also constitutes an important widely used prognostic marker [12]. The New York Heart Association (NYHA) classification system is a formal system for assessing the functional exercise capacity of a patient where a higher NYHA class (IV, III) is associated with an increase in experienced HF symptoms, a decreased quality of life, and poor survival [13-15]. This classification system is highly subjective [12,16], with low inter-rater reliability, especially for NYHA class II and III [17]. The application of the criteria, thus, varies widely based on the patients’ self-report and the individual physician’s interpretation [12,16]. A quantifiable measure that removes this subjectivity to make the assessment of NYHA class more repeatable and objective would be beneficial.

A previous exploratory study [18] investigated wearable activity trackers in patients with HF and demonstrated a statistically significant difference between the daily average step counts (a proxy for exercise intolerance) in patients exhibiting NYHA class II and III symptoms. However, the study’s small sample (n=8) limits scientific confidence in the generalizability of these findings. The primary aim of this study was to determine if these findings can be replicated using a larger sample collected independently from the original pilot study data. A secondary aim was to investigate wearable activity tracker usage by patients with HF and begin to characterize the step count distribution of these patients under free-living conditions in hopes of enabling the engineering of objective methods of assessing and monitoring NYHA functional class and, thereby, improving the ability of clinicians to accurately assess disease progression and severity.

## Methods

### Ethics Approval

This study is covered by institutional and research ethics approval (REB #14-7595) received from the University Health Network REB; (signed) informed consent was obtained from all study participants.

### Recruitment

Patients in a moderately larger dataset (n=50) were consecutively recruited, as part of a broader umbrella study, from the Heart Function Clinic at Toronto General Hospital (TGH) in Toronto, Canada, from September 2014 to June 2015. The inclusion and exclusion criteria used are outlined in Textbox 1.

**Study inclusion and exclusion criteria.**

**Inclusion criteria:**

- Adults (aged older than 18 years)
- Stable chronic heart failure
- New York Heart Association class II or III
- Left ventricular ejection fraction ≤35% (arising out of research requirements for the broader umbrella study)
- Able to walk without walking aids
- Capable of undergoing consent, understanding English instructions, and complying with the use of the study devices.

**Exclusion criteria:**

- Congenital heart disease
- Diagnosis less than 6 months before recruitment
- Traveling out of Canada for more than 1 week during the study period (to limit study costs–ie roaming charges)

#### Data Collection

Patients were supplied with a Fitbit Flex [19], an Android smartphone (Moto-G), the associated charging equipment for both devices, as well as a cellular internet data plan to facilitate syncing the tracker to the Fitbit server. Patients were instructed to wear the Fitbit daily on the same wrist, preferably their nondominant hand, for a period of 2 weeks, except during water activities such as showering or swimming, as the Flex is not waterproof. Patients were also instructed to charge the Fitbit at least every 3 days, preferably while they slept. The Fitbit data were retrieved using an open-source script published and available on GitHub and adapted for this study [20].

#### Population

Patients in our larger dataset were labeled as either NYHA class II and III or (according to standard practice in our clinic) when a physician was uncertain about the classification or felt that patients exhibited symptoms from different class levels, as a borderline or mixed class: I/II or II/III. As NYHA class I/II and II/III are not formally recognized NYHA classes, to perform our analysis, the authors regrouped borderline patients into one of the traditional 4 class NYHA according to the most extreme NYHA class in the mix and according to the following rationale: as NYHA class I corresponds to “no limitation of physical activity,” [15] an absolute binary (yes/no) distinction, a patient assigned as class I/II, who necessarily must be exhibiting strictly more than “no limitation of physical activity” [15] (however slight) can be reasonably grouped with class II patients generally (those exhibiting “a slight limitation of physical activity”) [15]. We designated this class I/II and class II group as NYHA group II^*^.

We extended the same line of reasoning for II/III patients, noting that patients assigned as class II/III must have experienced some *more* marked limitation of physical activity beyond that seen in patients classified in class II. As such, for consistency, we grouped them with the lower class III. We designated this class II/III and III group as NYHA group III*.

### Statistics

**Figure 1:**
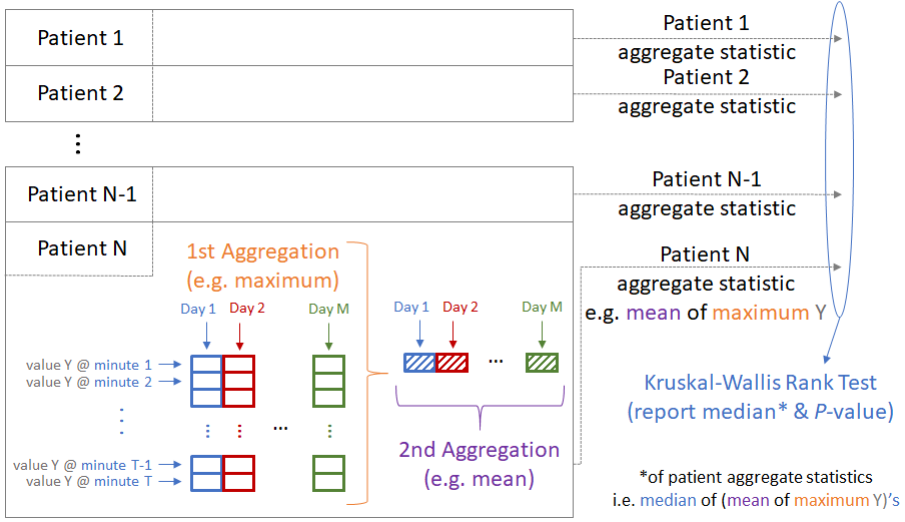
Summary Statistic Computation Methodology.

**Figure 2:**
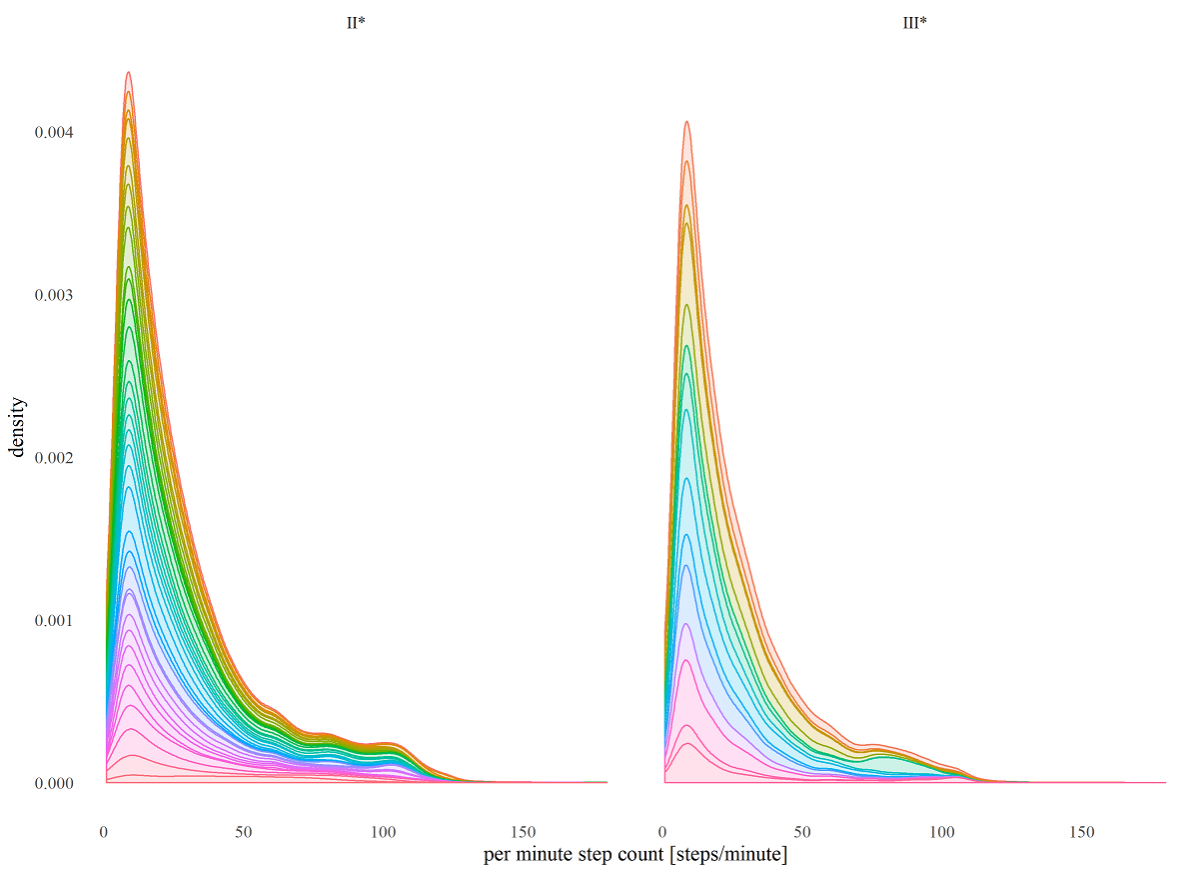
Combined (all patients) distribution of per minute step counts by NYHA group (only step count values > 0). Colored internal segments illustrate relative contributions to distribution by each study participant.

Consistent with our previous study [18], we used the Kruskal-Wallis rank test to compare the experimental variables of interest, including the mean daily total step count. As the data are clearly not normally distributed—as can be seen in Figure 1—and in keeping with the secondary aim of the study, we also computed various additional statistical summaries of the minute-by-minute step count data to attempt to better characterize the data distribution. To calculate these summaries, we performed a first aggregation: calculating statistical summaries (mean, SD; 5-number summaries; interquartile range [IQR]; skewness; and kurtosis) across each patient’s individual patient-day of step data and then a second aggregation across the day summaries, calculating the max, min, mean, and SD of each patient’s daily summaries for the 2-week period (producing a maximum of mean daily step counts, minimum of mean daily step counts, and mean of mean daily step counts) to assess overall variation across patient-days. The methodology is shown graphically in Figure 2. In addition, we generated statistical summaries treating the overall 2-week period as 1 continuous time period (instead of analyzing it day-by-day) and simply performed a single (1st) aggregation over that period to generate the corresponding statistical summary for that patient-period. We then performed a Kruskal-Wallis rank test on each of the generated statistical summaries and reported the corresponding median value of each NYHA group and the calculated unadjusted *P* value from the statistical test. Note that as we report unadjusted *P* values (ie, without multigroup correction), statistical significance should be interpreted in light of this limitation; rejection of the null hypothesis (ie, rejecting group II* statistical summary X=group III* statistical summary X) is, therefore, limited to that statistical summary alone—that is, in isolation from the other statistical tests performed. The analysis was performed using R [21], RStudio [22] with supporting packages [23-28].

## Results

Table 1 provides demographic information for each of the patients in the dataset according to their NYHA class. Table 2 provides demographic information for the overall dataset and for patients when the dataset is regrouped according to the labeling scheme described in the Methods section (Population subsection). The patients are predominantly male (83% vs 93%), aged (median [IQR]): 55 (19) vs 56 (18) years, and overweight (body mass index (median [IQR]): 27.1 (7.6) vs 29.6 (6.6) kg/m^2^).

**Figure 3:**
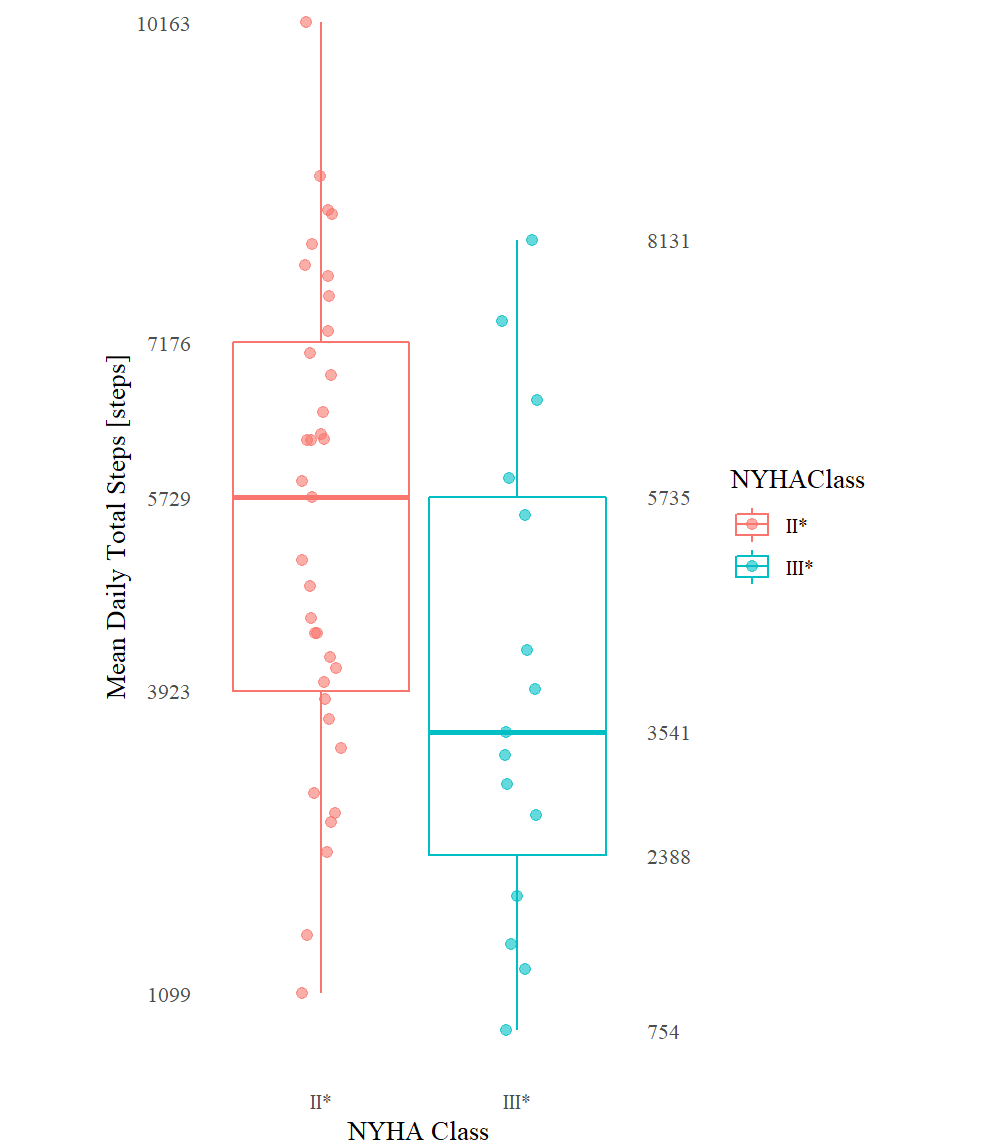
Boxplots (min, Q1, median, Q3, max) of mean daily total steps for each NYHA class group.

**Figure 4:**
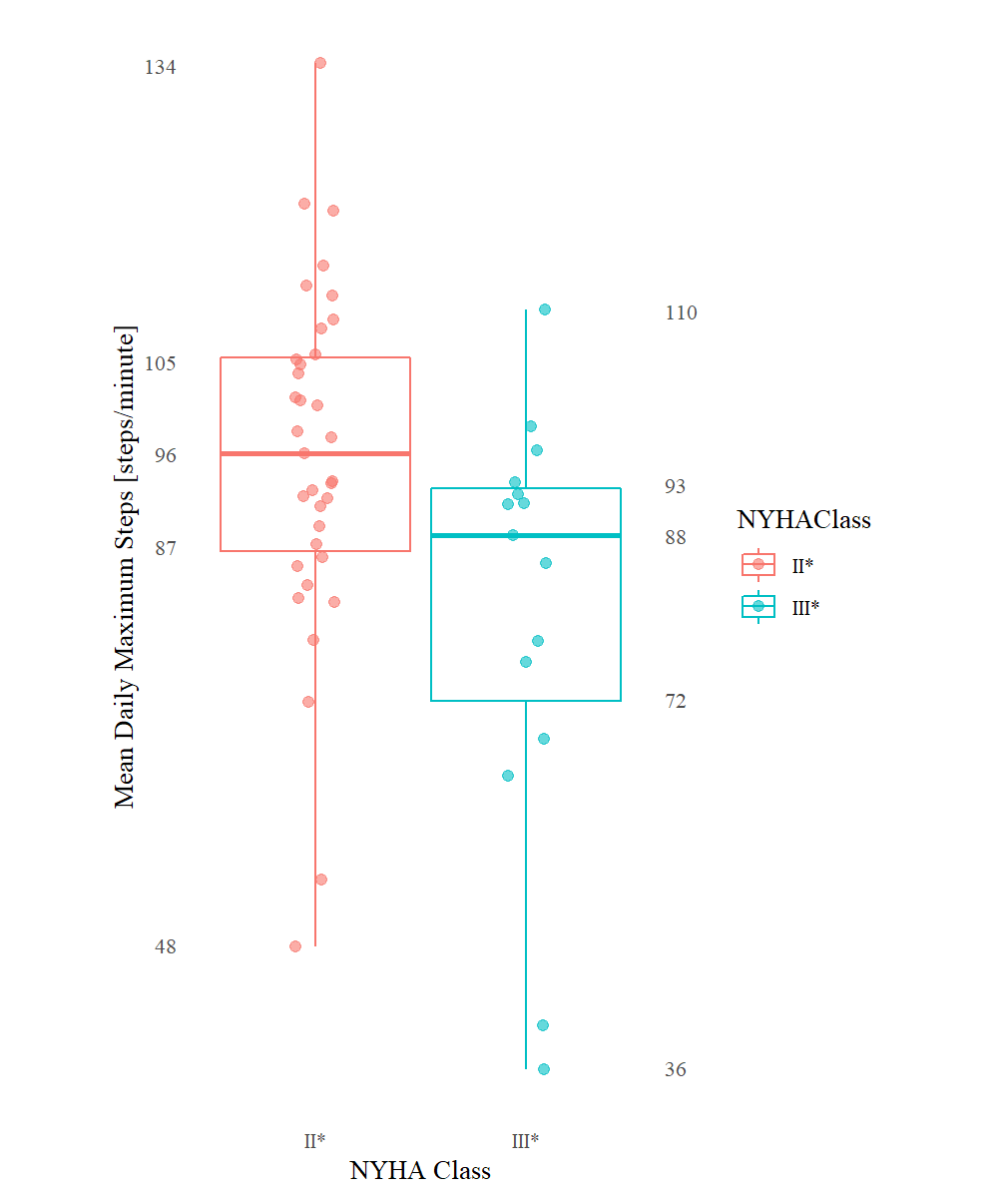
Boxplots (min, Q1, median, Q3, max ) of mean daily per minute step count maximums for each NYHA class group.

**Figure 5:**
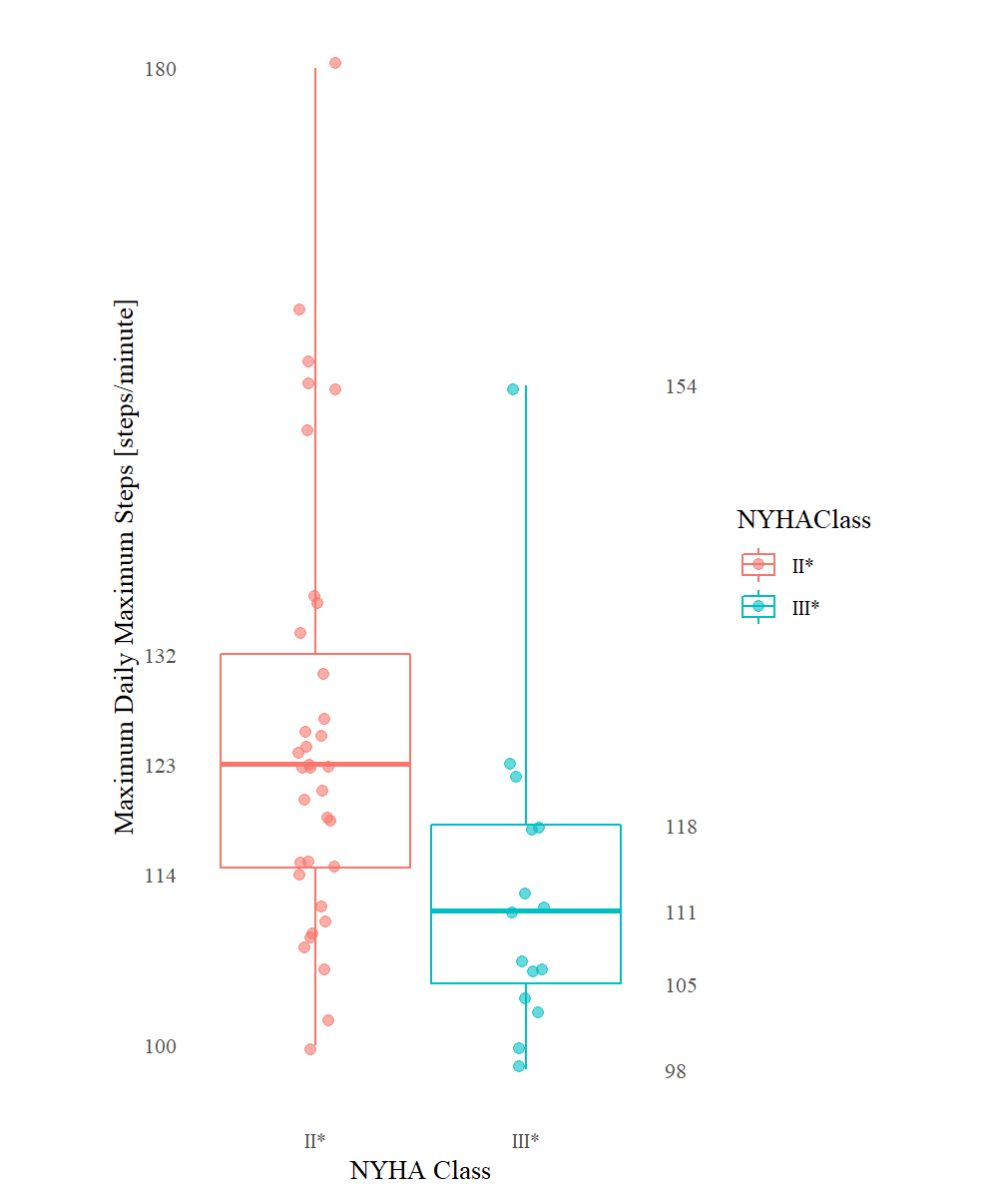
Boxplots (min, Q1, median, Q3, max ) of maximum daily per minute step count maximums for each NYHA class group.

Table 3 includes results that were found to be significant at the *P*=.05 level of significance (reported as median values because of the use of the nonparametric Kruskal-Wallis rank test). Table 4 contains the remaining nonsignificant results excluding any statistical summary that returned a 0 value for all classes (eg, aggregations involving daily or overall minimum, 1st, 2nd, and 3rd quartile) because of the overwhelming frequency of 0 per minute step count. The mean daily total steps and the mean and max of daily per minute step count maximums are plotted graphically in Figures 3, 4, and 5, respectively.

**Table 1:** Study dataset demographics (by original New York Heart Association class label).

Variable	NYHA^a^ I/II	NYHA II	NYHA II/III	NYHA III
Participants, n (%)	9 (18)	26 (52)	4 (8)	11 (22)
Number of males, n (%)	6 (67)	23 (89)	4 (100)	10 (91)
Age (years), Q1^b^|M^c^|Q3^d^	50|52|62	45|57|66	45|50|56	53|58|68
Height (cm), Q1|M|Q3	167|172|180	167|172|180	167|172|180	167|172|180
Weight (kg), Q1|M|Q3	60.0|84.8|96.0	79.0|84.5|93.8	80.8|96.2|103.8	82.0|94.0|104.0
BMI (kg/m^2^), Q1|M|Q3	21.5|24.0|29.3	25.0|27.6|31.7	25.8|30.4|33.0	27.0|29.6|32.8

^a^NYHA: New York Heart Association.

^b^Q1: 1st quartile.

^c^M: median.

^d^Q3: 3rd quartile.

**Table 2:** Study regrouped dataset demographics (Overall, New York Heart Association group II^*^ and III^*^).

Variable	Overall	NYHA^a^ Group II^*^	NYHA Group III^*^
Total participants, n (%)	50 (100)	35 (70)	15 (30)
Number of males, n (%)	43 (86)	29 (83)	14 (93)
Age (years), Q1^b^|M^c^|Q3^d^	47|55|65	46|55|65	49|56|67
Height (cm), Q1|M|Q3	170|175|180	168|175|179	171|177|180
Weight (kg), Q1|M|Q3	74.9|89.0|96.5	73.4|84.8|95.0	82.0|94.0|104.2
BMI (kg/m^2^), Q1|M|Q3	24.7|28.1|32.1	24.0|27.1|31.6	27.0|29.6|33.6

^a^NYHA: New York Heart Association.

^b^Q1: 1st quartile.

^c^M: median.

^d^Q3: 3rd quartile.

**Table 3:** Significant findings for comparisons between group II* and group III*.

Variable		Group II^*^ (=I/II+II), median	Group III^*^ (=II/III+III), median	*P* value
**Maximum**				
	Maximum 2-week PMSC^a^ (steps/minute)	123.0	111.0	.004^b^
	Maximum of maximum DPMSC^c^ (steps/minute)	123.0	111.0	.004^b^
	Mean of maximum DPMSC (steps/minute)	96.1	88.1	.02^d^
**Mean**				
	Mean 2-week PMSC (steps/minute)	4.0	2.5	.04^d^
	Maximum of mean DPMSC (steps/minute)	7.5	4.1	.03^d^
	Mean of mean DPMSC (steps/minute)	4.0	2.5	.04^d^
	SD of mean DPMSC (steps^2^/minute^2^)	1.8	1.1	.04^d^
**SD**				
	SD of 2-week PMSC (steps^2^/minute^2^)	13.3	9.2	.02^d^
	Maximum of DPMSC SD (steps^2^/minute^2^)	20.6	14.5	.002^b^
	Mean of DPMSC SD (steps^2^/minute^2^)	12.0	8.8	.03^d^
**Total**				
	Total 2-week SC^e^ (steps)	88130	53123	.03^d^
	Maximum of total DPMSC (steps)	10792	5904	.03^d^
	Mean of total DPMSC (steps)	5729	3541	.04^d^
	SD of total DPMSC (steps^2^)	2570	1513	.04^d^

^a^PMSC: per minute step count.

^b^*P*<.01.

^c^DPMSC: daily per minute step count.

^d^*P*<.05.

^e^SC: step count.

**Table 4:** Nonsignificant findings for comparisons between group II* and group III*.

Variable		Group II^*^ (=I/II+II), median	Group III^*^ (=II/III+III), median	*P* value
**Demographics**							
	Sex (male=0, female=1)	0	0	.33
	Age (years)	55	56	.71
	Height (cm)	175.0	177.0	.38
	Weight (kg)	84.8	94.0	.17
	BMI^a^ (kg/m^2^)	27.1	29.6	.28
	Righthanded?^b^ (no=0, yes=1)	1	1	.18
	Wristband preference^c^ (left=0, right=1)	0	0	.16
**Maximum**							
	SD of maximum DPMSC^d^ (steps^2^/minute^2^)	24.6	23.5	.76
	Minimum of maximum DPMSC (steps/minute)	42.5	34.7	.58
**75th percentile**							
	Maximum of 75th percentile of DPMSC (steps/minute)	0	0	.93
	Mean of 75th percentile of DPMSC (steps/minute)	0	0	.89
	SD of 75th percentile of DPMSC (steps/minute)	0	0	.91
**Mean**							
	Minimum of mean DPMSC (steps/minute)	0.3	0.1	.90
**Median**							
	Median of 2-week PMSC^e^ (steps/minute)	0	0	N/A
	Maximum of median DPMSC (steps/minute)	0	0	N/A
	Minimum of median DPMSC (steps/minute)	0	0	N/A
**Total**							
	Minimum of total DPMSC (steps)	420	164	.90
**Interquartile range (IQR)**							
	Maximum of DPMSC IQR (steps/minute)	0	0	.93
	Mean of DPMSC IQR (steps/minute)	0	0	.89
	SD of DPMSC IQR (steps^2^/minute^2)^	0	0	.91
**SD**							
	Minimum of DPMSC SD (steps^2^/minute^2^)	2.9	1.2	.80
**Skewness**							
	2-week PMSC skewness	4.6	5.5	.29
	Maximum of daily SC^f^ skewness	8.8	8.5	.97
	Mean of daily SC skewness	4.9	5.1	.76
	SD of daily SC skewness	1.3	1.4	.76
	Minimum of daily SC skewness	3.3	3.4	.65
**Kurtosis**							
	2-week PMSC kurtosis	24.5	36.0	.25
	Maximum of daily SC kurtosis	99.3	99.4	.97
	Mean of daily SC kurtosis	31.7	33.4	.71
	SD of daily SC kurtosis	20.1	22.8	.73
	Minimum of daily SC kurtosis	10.4	13.2	.47

^a^BMI: body mass index.

^b^Is patient righthanded?

^c^Right- or lefthanded preference for wristband.

^d^DPMSC: daily per minute step count.

^e^PMSC: per minute step count.

^f^SC: step count.

## Discussion

### Principal Findings

This study, using an independent, larger group of participants, replicated and validated the findings of our previous pilot study: that the daily free-living step counts of patients with HF exhibiting NYHA class II versus class III symptoms (ie, group II* vs group III*) are statistically different [18].

Specifically, HF patients categorized as NYHA II^*^ and III^*^ differed significantly (at the 5% level of significance) in their mean of daily total step counts (group medians: 5729 vs 3541; *P*=.04), maximum of daily total step counts (10792 vs 5904; *P*=.03), mean of daily mean step counts (4.0 vs 2.5; *P*=.04), as well as by their mean (96.1 vs 88.1; *P*=.02) of daily per minute step count maximums. These same patients differed significantly (at the .01% level of significance) by their maximum of daily per minute step count maximums (123.0 vs 111.0; *P*=.004, respectively). The distribution of the per minute step counts by NYHA class—including only all nonzero per minute step count values—is shown in Figure 1. The daily step count results mimicked the 2-week overall step count values.

A total of 10,000 (steps/day) is often recommended as the daily step target for healthy adults, although in practice “many people can only achieve about slightly more than half of the daily step goal” with a meta-analysis of studies revealing ranges between 5300 and 6700 daily steps [29]. Persons who average <5000 (steps/day) are considered to be living a sedentary lifestyle, with persons averaging between 5000 to 7499 (steps/day) living a “low active” lifestyle [30,31]. Ayabe et al, based on a study of 77 cardiac rehab patients aged 46 to 88 years, recommended daily step targets of 6500 to 8500 (steps/day) for the secondary prevention of cardiovascular disease [32]. The NYHA group II* patients in our study, whose group median was a grand mean of 5729 (steps/day), achieved what would be considered a “low active” lifestyle near the bottom of the average daily step range of healthy adults and below the prevention target. In contrast, the NYHA group III* patients in our study, with a grand mean of 3541 (steps/day) (group median), fell well within the “sedentary” lifestyle range, well below the expected average daily step range of healthy adults and well below the secondary prevention target. Furthermore, at their peak within the 2-week study period—indicated by the maximum daily per minute step count total of 5904 (steps/day; group median)—the NYHA group III* patients never exceeded the “low active” lifestyle range neither did they come near to achieving the secondary prevention target, let alone the 10,000 (steps/day) target. In fact, at their peak, over the 2 weeks, the NYHA group III*’s maximum daily step count (group median: 5904 [steps/day]) only barely exceeded group II*’s grand mean step count (group median: 5729 [steps/day]). The NYHA group II* in comparison achieved a maximum daily per minute step count total of 10,792 (steps/day; group median): above both the secondary prevention target and the 10,000 (steps/day) target. Taken together, these numbers appear to quantitatively demonstrate a “marked limitation of physical activity” for patients with NYHA class III compared with a more “slight limitation of physical activity” for patients with NYHA class II (both corresponding to their respective NYHA functional classification criteria [17]).

As for the general shape of the step count distributions of the NYHA group II* versus III* patients, visual inspection of Figure 1 strongly suggests that there is a difference in the activity patterns of patients, for example, a longer, fatter tail for the group II* patients. Quantitatively, however, we failed to extract many meaningful insights into the shape of the activity distribution. The 1st, 2nd, and 3rd quartile (and by extension IQR), for example, were all found to be fairly consistently 0 for all patients, that is, 0’s typically accounted for more than 75% (1,080/1,440) of the data points for any given patient day. This is because, unfortunately, the activity tracker used in this study records 0’s both when a patient is not active and when the patient is simply not wearing the tracker. Not only does this make it difficult to ascertain if a 0-step count indicates lack of activity or patient’s lack of adherence but it also means that we are unable to remove the excess 0’s introduced into the apparent distribution as a result of a participant’s lack of adherence to the tracker.

In light of the challenge introduced by the tracker selection, it is curious that in comparing the step count intensity measures (ie, maximum and mean daily aggregated per minute step count), the maximum daily per minute step count maximum values for each patient group was found to be notably more statistically significant compared with the other intensity measures. Of course, because of the nature of the statistic, metrics involving maximums would naturally be least susceptible to the ambiguous 0 per minute step count values. We suggest that this may be contributing to the daily maximum values appearing as more strongly differentiating between the 2 NYHA groups.

There are, however, other explanations for the phenomenon detailed above, including differences in accuracy of activity trackers at different step-intensity levels. Activity trackers, including the Fitbit, have been shown to be sufficiently accurate for research purposes [33-39]; however, several researchers have reported a degradation of accuracy in these devices (including the Fitbit Flex used in this study) at low and medium step cadences [35,37,39]. For example, An et al found that the accuracy of the device used in our study varied between 6.2% and 11.4% at the low and medium treadmill speeds (2 to 4 mph) that they tested but improved to 4% at the highest speed tested (5 mph) [37]. It is possible, therefore, that the more accurate recordings at high intensity levels simply makes it more possible to differentiate between the step counts of patients in each group regardless of the effect of superfluous 0-step count values.

Alternatively, it is also reasonable that the overall step count maximum, by capturing a patient’s peak exercise capacity, might produce a more reliable (detectable) measure of the “limitation of physical activity” experienced by a patient in daily life and thus help differentiate more consistently between NYHA classes (compared with a simple mean or sum of a patient’s activity over a said day). For example, previous in-laboratory studies observing patients performing a 6-minute walk test have been found that, on average, patients with the relatively higher NYHA class II spend more time (56%) at higher step intensities (>120 steps/minute) compared with patients with NYHA class III (24% of overall time) and vice-versa at lower step intensities (12% vs 36% of overall time at <100 steps/minute) [34]. It might just be that peak exercise generally may simply be a more consistent way of gaining insight into a patient’s NYHA class than their average activity level.

### Strengths and Limitations

A major limitation of this study is the grouping methodology used to reclassify patients who are assigned a borderline/mixed NYHA class to make them fit within the traditional 4-class NYHA classification system. The approach we used, although logically reasonable, has no demonstrated scientific support. Furthermore, the data being sourced as a convenience sample at the same single site, that is, consecutively recruited from the TGH Heart Function, represent a limitation of this study with regard to our objective of generalizing our findings. Our analysis was also limited as it did not include any patients with NYHA class I or IV. Although these are not typically as difficult to classify as NYHA class II or III patients, analysis of all 4 NYHA classes would have potentially provided additional useful insight into the true underlying relationship between step count and NYHA class. Knowing exactly how step count and NYHA class are related may be tremendously valuable if it allows us to assess or predict NYHA class or gradation changes in NYHA class for a patient using their step count. We suggest that this might be the subject of an important future study.

**Figure 6:**
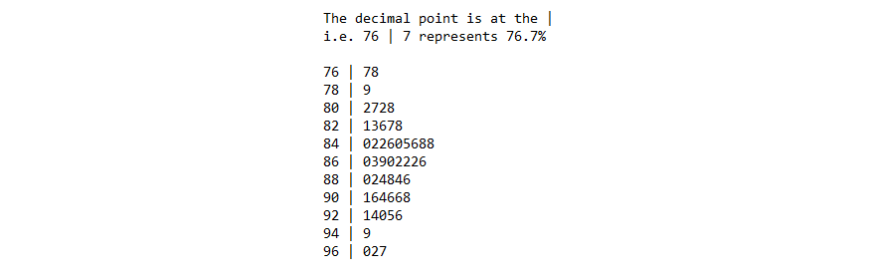
Number of zero step count minutes as a percentage of the total two-week data stream for each patient.

The most significant limitation of our study, however, was the step tracker utilized, as it introduced significant ambiguity into the 0 per minute step count values which comprised most of each patient’s step data stream. Zero values accounted for a mean of 87.3% (SD 4.9%) of the 2-week data stream for each patient—accounting for as much as 97.7% of the 2-week data stream for one of the patients. In fact, when looking at the 2-week period as a whole, they accounted for at least 76.7% of all the data points for any given patient. The complete breakdown is shown in Figure 6. Unfortunately, the meaning of these 0 per minute step count values is ambiguous as the trackers used in this study record a 0 value not only during patient inactivity—for example, when a patient is sitting, sleeping, or generally not moving—but also when the patient was simply not wearing the device—for example, to charge it. As a result, it is challenging to accurately determine if a given series of zeroes indicates a pattern of low physical activity—presumably explanatory of an NYHA class—or simply a pattern of no device usage—essentially introducing noise into the physical activity signal. This limits our ability to precisely quantify the distribution of the activity/inactivity of patients, especially as it is as of yet unclear how much importance patient inactivity (vs patient activity) should be accorded when it comes to capturing “physical activity limitation” and by extension the NYHA functional class. Investigations into how to disambiguate between inactive versus disengaged/nonadherence time in pedometer-like trackers would be hugely beneficial to help researchers correct for the effect of nonadherent time in the captured free-living step data distribution, especially if we are to better understand the actual true relationship between free-living activity and NYHA functional classification. At the very least, we recommend that future researchers strongly consider using an activity tracker that clearly disambiguates between inactivity and patient disengagement or provides an additional data stream that would support some reliable objective means of performing the disambiguation.

### Conclusions

On average, patients exhibiting NYHA II versus NYHA III symptoms are expected to exhibit “low active” versus “sedentary” lifestyles with (1) mean daily step count totals around 5729 (steps/day) versus 3541 (steps/day; group medians)—in the case of patients exhibiting NYHA III symptoms less than the 5300 to 6500 (steps/day) expected of typical healthy adults and in the case of patients exhibiting NYHA II symptoms only barely within the same range and (2) maximum daily step count totals of 5904 versus 10,792 (steps; II vs III group medians)—compared with the healthy target of 10,000+ average (steps/day). These findings validate our previous pilot study and point to limitations in daily physical activity beyond those found in normal healthy adults. In addition, consistent with laboratory tests, patients exhibiting NYHA class III symptoms are on average expected to exhibit lower step count intensities during free-living with (group medians II vs III) (1) mean (2-week) daily mean step counts of 4.0 versus 2.5 (steps/minute), (2) a mean daily per minute step count maximums of 88.1 versus 96.1 (steps/minute), and (3) a maximum daily per minute step count maximums of 111.0 versus 123.0 (steps/minute).

The discovery of additional significant aggregate measures raises several questions, among them are the following: What is the exact underlying relationship between NYHA functional class and step count? What features of the step count waveform are most associated or correlated with NYHA functional class? These questions will no doubt feature as the subjects of future studies, but the findings of this study are an important milestone on the road to an objective means of assessing HF functional classification on our continuing quest to improve outcomes of patients with the burdensome and costly disease, that is, congestive HF.

## Abbreviations

CHD: coronary heart disease
EF: ejection fraction
HF: heart failure
HFpEF: heart failure with preserved ejection fraction
HFrEF: heart failure with reduced ejection fraction
HT: hypertension
IQR: interquartile range
LVEF: left ventricular ejection fraction
NYHA: New York Heart Association
TGH: Toronto General Hospital
